# Role of Ceramides in the Molecular Pathogenesis and Potential Therapeutic Strategies of Cardiometabolic Diseases: What we Know so Far

**DOI:** 10.3389/fcell.2021.816301

**Published:** 2022-01-19

**Authors:** Youssef M. Shalaby, Anas Al Aidaros, Anjana Valappil, Bassam R. Ali, Nadia Akawi

**Affiliations:** ^1^ Department of Genetics and Genomics, College of Medicine and Health Sciences, United Arab Emirates University, Al-Ain, United Arab Emirates; ^2^ Department of Pharmacology and Toxicology, Faculty of Pharmacy, Ahram Canadian University, Egypt; ^3^ Zayed Centre for Health Sciences, United Arab Emirates University, Al-Ain, United Arab Emirates; ^4^ Division of Cardiovascular Medicine, Radcliffe Department of Medicine, University of Oxford, Oxford, United Kingdom

**Keywords:** ceramides, cardiometabolic diseases, ROS, cytokines, apoptosis

## Abstract

Ceramides represent a class of biologically active lipids that are involved in orchestrating vital signal transduction pathways responsible for regulating cellular differentiation and proliferation. However, accumulating clinical evidence have shown that ceramides are playing a detrimental role in the pathogenesis of several diseases including cardiovascular disease, type II diabetes and obesity, collectively referred to as cardiometabolic disease. Therefore, it has become necessary to study in depth the role of ceramides in the pathophysiology of such diseases, aiming to tailor more efficient treatment regimens. Furthermore, understanding the contribution of ceramides to the pathological molecular mechanisms of those interrelated conditions may improve not only the therapeutic but also the diagnostic and preventive approaches of the preceding hazardous events. Hence, the purpose of this article is to review currently available evidence on the role of ceramides as a common factor in the pathological mechanisms of cardiometabolic diseases as well as the mechanism of action of the latest ceramides-targeted therapies.

## Introduction

Cardiometabolic disorders is an umbrella term for a group of interrelated diseases and risk factors including cardiovascular diseases (CVDs), type II diabetes, hypercholesterolemia, and their underlying risk events such as insulin resistance, endothelial dysfunction, and atherosclerosis (Zhang et al., 2018; Miranda et al., 2019; Yang et al., 2021). Researchers are constantly searching for new biomarkers to help in the early diagnosis of such diseases and ways for addressing the increasing levels of their prevalence across the world (Roberts and Gerszten, 2013). Out of these diseases, CVDs remain one of the world’s biggest killers to mankind, despite the significant advancements in related therapies (Goradel et al., 2018). CVDs impose a devastating and crippling economic impact on health care systems globally as the direct costs of CVDs surpass medical costs for any other chronic condition (Go et al., 2014). Therefore, it is generally accepted that new therapeutic solutions and prognostic biomarkers are urgently needed to reduce the suffering of patients with CVDs and health care costs (Deng et al., 2020).

Excitement and hopes flared when researchers found a correlation between ceramides levels and prevention of metabolic CVDs in animal models (Bikman and Summers, 2011a; Raichur et al., 2014). Later, researchers found that increased levels of circulatory ceramides in humans resulted in their accumulation in various types of tissues, particularly adipose tissue, which may have beneficial or pathological consequences on their health depending on the type of ceramide (Summers et al., 2019). For example, the length of either sphingoid or N-acyl chain has been found to be a determinant factor for ceramides physiological and pathological properties as well as their synthesis (Alonso and Goñi, 2018). Indeed, although ceramides at normal levels have useful physiological and biological functions, such as reducing the concentration of free fatty acids through facilitating fat storage, their abnormal levels can impair the cardiovascular system in addition to the induction of obesity-related metabolic complications such as insulin resistance, atherosclerosis, and liver diseases (Summers, 2006). Given the fact that a variety of human diseases such as CVDs, diabetes and neurological diseases have become coupled with the circulating levels of ceramide, it has been proposed to use ceramides as reliable biomarkers for the prediction of such pathological conditions (Kurz et al., 2019). Using Liquid chromatography-tandem mass spectrometry (LC-MS/MS) has enabled researchers to measure the normal plasma levels of ceramides in adult populations and reference ranges were determined to be 0.26–0.34 μmol/L for C16:0 ceramide, 0.09–0.14 μmol/L for C18:0 ceramide, and 0.96–1.35 μmol/L for C24:1 ceramide (Meeusen et al., 2018). Based on the plasma concentration of ceramides (C16:0, C18:0, C24:1) and their ratios to C24:0 ceramide, a risk of developing CVDs can be categorized according to standardized risk scores (e.g., CERT1 score) into low, moderate, increased and high risk (Carrard et al., 2021). However, numerous limitations of using LC-MS/MS to measure ceramides still exist particularly regarding its specificity in detecting the broad spectrum of ceramides species and derivatives, in addition to the substantial variation of these lipids among individuals depending on many factors such as age, sex, and diet (Gaggini et al., 2021).

## Ceramides Structure and Physiological Function

**FIGURE 1:**
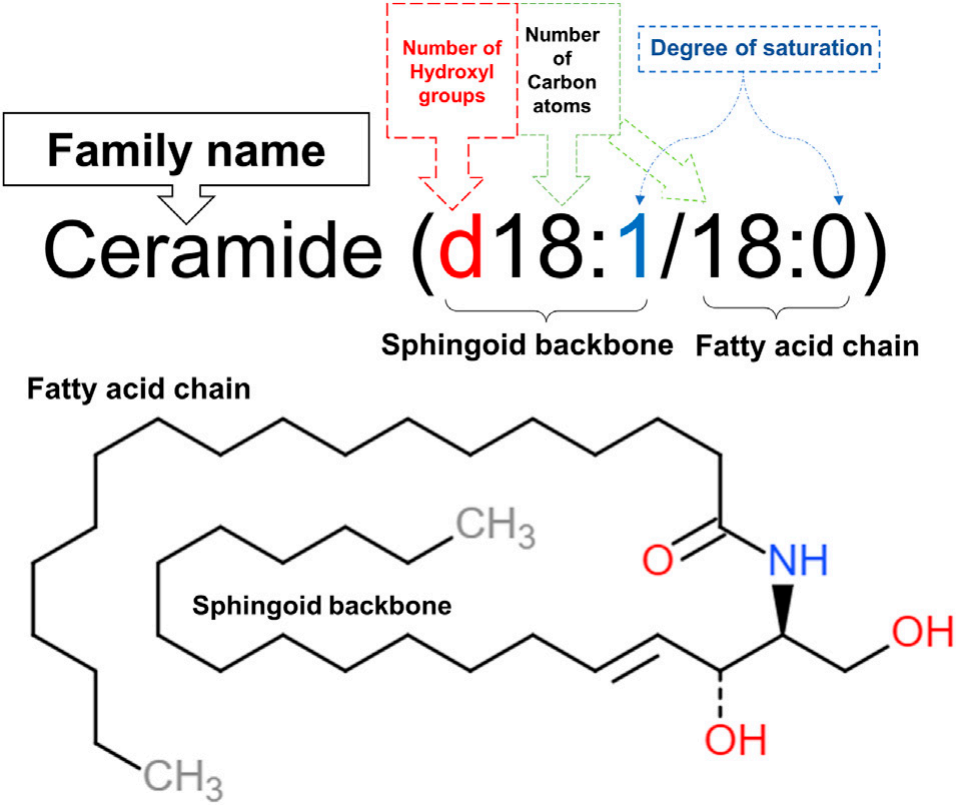
Nomenclature of C18:0 ceramide (d18:1/18:0). The first part of the name (d18:1) denotes the 18 carbon atoms, having one double bond in its sphingoid backbone along with two hydroxyl groups. This sphingosine chain is attached to a saturated fatty acid chain, represented by the second part of the name (18:0), through an amide bond. This illustration was adapted from caymanchem.com.

Structurally, ceramides belong to sphingolipids as they individually have a long-chain fatty acid (non-hydroxy acids, α-hydroxy acids and ω-hydroxy acids), that is amide-linked to a sphingoid base (Figure 1), namely sphingosine, phytosphingosine, dihydrosphingosine or 6-hydroxysphingosine. In human skin, over a hundred ceramide subclasses have been identified so far (Wartewig and Neubert, 2007; Alonso and Goñi, 2018). Ceramides exist mainly as structural elements in cell membranes since they are derived from sphingolipids that make up sphingomyelin, a major component of the phospholipid bilayer. Besides their structural function, ceramides play significant roles in cell signaling as they act as second messengers modulating several metabolic pathways depending on their chain length (Grösch et al., 2012). Furthermore, ceramides possess a central role in cell biological activities, including proliferation, differentiation, senescence, as well as inflammation, and apoptosis (Rivera et al., 2015). The nomenclature of ceramides relies on the number of carbon atoms in the sphingoid backbone, fatty acid chain, and saturation level (McGurk et al., 2021) as shown in Figure 1, naming C18:0 ceramide (d18:1/18:0) as an example.

## Ceramides Biosynthesis

Many studies have revealed some of the pivotal triggers for ceramide *de novo* synthesis. For instance, exogenous lipid overload, ultraviolet B rays (UVB), and cytokines can increase the expression of serine palmitoyltransferase (SPT), which in turn increases ceramide production. It is worth mentioning that these studies have found that TNF-α, Fas ligand, toll-like receptor-4 activation, or oxidative stress may increase the breakdown of sphingomyelin into ceramides, which has been described as a stress-activated pathway (Summers, 2006;Sokolowska and Blachnio-Zabielska, 2019; Chaurasia and Summers, 2020).

**FIGURE 2:**
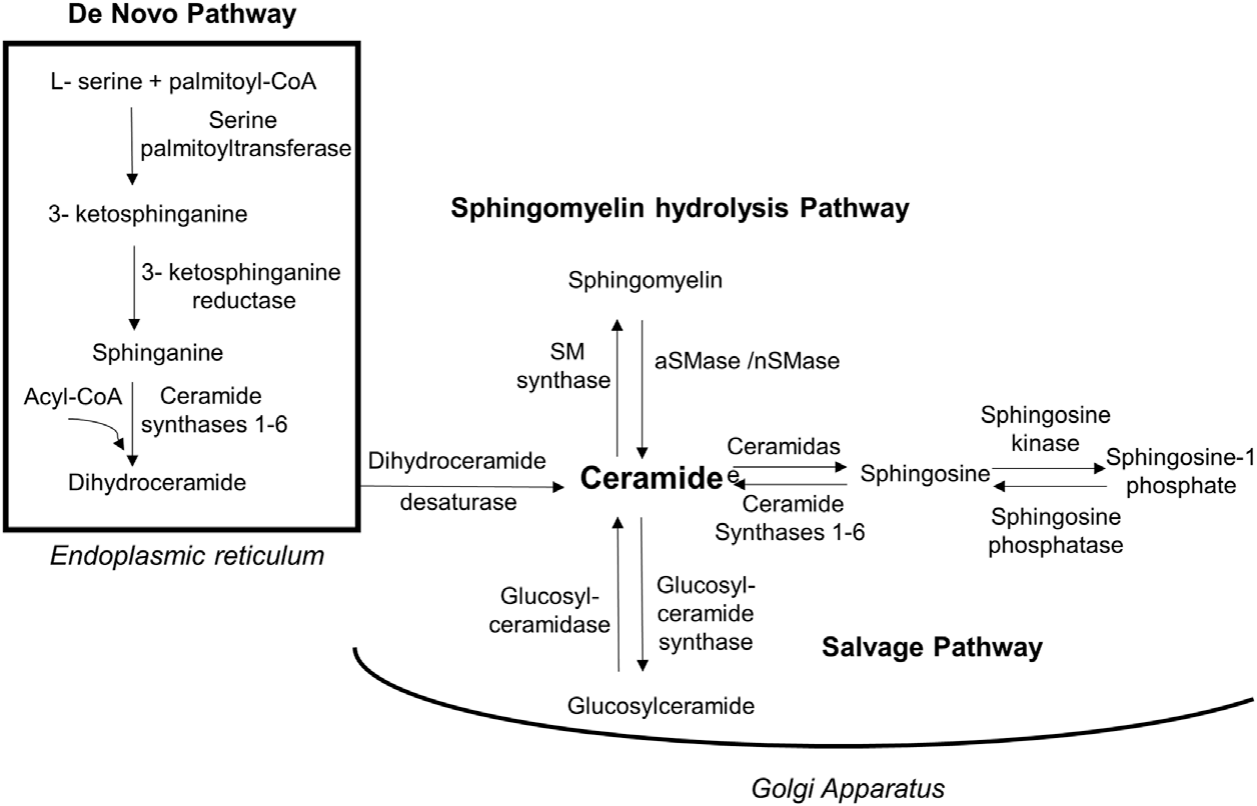
Biosynthesis routes of ceramides. Ceramides are synthesized via 3 pathways. Firstly, *de novo* synthesis of ceramides starts in endoplasmic reticulum by coupling amino acid serine to palmityl-CoA using palmitoyltransferase in a multi-step process and yielding dihydroceramide that will be activated into ceramide by dihydroceramide desaturase. Secondly, in Golgi apparatus ceramides can be generated by sphingomyelin hydrolysis via acidic/neutral sphingomyelinase. The third biosynthesis method is through salvage pathway in which glucosylceramide is converted into ceramides via glucosylceramidase. Moreover, ceramides can be metabolized into less toxic sphingosine that get phosphorylated to sphingosine-1phosphate via sphingosine kinase. [aSMase, acid sphingomyelinase; nSMase, neutral sphingomyelinase; SM, sphingomyelin].

In addition to the *de novo* synthesis pathway which is initiated by condensation of serine with palmitoyl-CoA via SPT, ceramides can be synthesized via two more different pathways as illustrated in Figure 2. These include the sphingomyelin pathway where hydrolysis of sphingomyelin to ceramide occurs using neutral or acid sphingomyelinase (nSMase or aSMase, respectively), and the salvage pathway, in which ceramides are recycled and generated from their metabolites sphingosine and glucosylceramide by ceramide synthase and glucosylceramide synthase, respectively (Sokolowska and Blachnio-Zabielska, 2019). The identification of many types of ceramides with various lengths of fatty acid side chains that exhibit different levels of saturation in human tissues facilitated our improved understanding of their chemistry, functions and pathophysiology (Moore and Rawlings, 2017). It has been stated that each of these types is initially produced as an intermediate dihydroceramide from sphinganine and C14-C30 acyl chain via dihydroceramide synthases, which include six protein isoforms family members (CerS 1-6). Thereafter, the intermediate is converted into a fully developed ceramide by dihydroceramide desaturase (Figure 2) (Cowart, 2009; Turpin et al., 2014).

The distribution of CerS enzymes among body tissues is variable (Table 1), where they catalyze the production of ceramides with different acyl chain lengths, and therefore with different functions. For instance, CerS1, CerS5, and CerS6 are widely distributed in skeletal muscle and brain tissues, and they are responsible for the generation of C16:0 and C18:0 long-chain ceramides (Grösch et al., 2012). In contrast, CerS2 which catalyzes the production of C20-C26 is abundant in many tissues, including heart, liver, and kidney tissues. It was reported that CerS enzymes are implicated in the regulation of various biological and metabolic functions through ceramide production in the human body. A prime example of those functions is that of CerS1-derived C18;0 ceramide, which is critical for brain development, while CerS2 resulting ceramides are essential for normal liver functions. However, it has been shown that dysregulation of CerS can lead to metabolic and CVDs (Raichur et al., 2014; Turpin et al., 2014).

**TABLE 1:** Ceramide synthases tissue distribution and their inhibitors as therapeutic targets.

Ceramide synthase (CerS)	Tissue distribution	Derived-ceramide	Physiological impact	Pathological alteration	Inhibitors	References
CerS1	C.N.S, skeletal muscle	C18:0	Brain development and neuronal signaling	Neurodegeneration (Parkinsonism), Insulin resistance	Myriocin	Levy and Futerman (2010); Abbott et al. (2014); Wegner et al. (2016); Choi et al. (2021)
CerS2	Brain, heart, Liver, kidney	C20:0- C26:0	Maintains healthy functions of lungs, brain, heart, and kidney tissues	Alzheimer disease, breast cancer, obesity, cardiomyopathy	S1P	Laviad et al. (2008); Wegner et al. (2016); Choi et al. (2021)
CerS3	Skin, testes	C22-C26	Spermatogenesis, normal keratinization	Disruption of skin barrier function	Not known	Park et al. (2014); Fucho et al. (2017)
CerS4	Heart, liver, skin	C18:0, C20:0	Stem cell homeostasis and hair growth	Obesity (m), diabetes(m), heart failure	ST1072	Laviad et al. (2008); Schiffmann et al. (2012); Abbott et al. (2014); Peters et al. (2015); Fucho et al. (2017); Choi et al. (2021)
CerS5	Heart, lungs, kidney, ubiquitous	C16:0	Brain development	Heart failure, apoptosis	Fingolimod (FTY720)	
CerS6	Heart, brain, other tissues	C14, C16:0	Brain development, immunity, tumor suppressor effects	Heart failure, obesity, multiple sclerosis	ST1072	Schiffmann et al. (2012); Park et al. (2014); Fucho et al. (2017); Choi et al. (2021)

C.N.S, central nervous system; m, mouse model; S1P, sphingosine 1 phosphate.

## The Interrelated Role of Ceramides in the Molecular Pathogenesis of Cardiometabolic Disorders

Recently, compelling evidence has been established regarding the contribution of ceramides to the molecular pathogenesis of CVDs along with the associated comorbidities through an interconnected mechanism (Ormazabal et al., 2018). Indeed, in addition to confirming the correlation between ceramides levels in the plasma and the risk of CVD, several large cardiac-cohort studies suggested ceramides as powerful prognostic biomarkers of CVDs progression in humans (Yu et al., 2015; Cao et al., 2020; Li et al., 2020; Akawi et al., 2021). Indeed, several studies specified three particular ceramides (C16:0, C18:0, C24:1) to be strongly associated with CVDs major adverse outcomes including cardiac related mortality (Fabbri et al., 2016; Li et al., 2020). For instance, in their comprehensive review, Cogolludo et al searched for human studies that evaluated the association of plasma levels of ceramides with adverse cardiovascular events and found 8 studies within their search scope. In 6 out of 8 reviewed studies, C16:0, C18:0, and C24:1 ceramides were found to be significantly linked to an increased risk of adverse cardiovascular outcomes and therefore considered to be the strongest predictive markers, among ceramides, for CVD (Cogolludo et al., 2019).

### Cardiac: Dilation and Contractility

In animal models, it has been suggested that alteration of ceramide signaling may contribute to the pathophysiology of diabetic cardiomyopathy (Colligan et al., 2002). In another *in vitro* study, ceramide (C2:0) has been shown to reduce high glucose-induced myocyte dysfunction, increase calcium influx, and improve smooth muscle contraction (Relling et al., 2003). On the other hand, ceramide analog dihydroceramide (C2:0) was reported to potentiate cardiac depressive effects of leptin, leading to cardiac dysfunction (Ren and Relling, 2006). Likewise, Javaheri et al have noted that elevated concentrations of circulating C16:0 and C18:0 ceramides were very much associated with the incidence of heart failure and that was attributed to CerS regardless of food intake (Javaheri et al., 2020). Prior to that, Bielawska et al. reported that a synthetic analogue of C16:0 ceramide induced apoptosis in cardiomyocytes of an ischemia-reperfusion rodent model (Bielawska et al., 1997). Furthermore, in animal models of obesity, ceramides were considered cardiotoxic molecules as they have contributed to the development of dilated cardiomyopathy as well as inhibition of cardiac contractility (Simon et al., 2014), which is consistent with previous observations of ceramide accumulation in CVD (Alewijnse et al., 2004). In humans, it was found that levels of long-chain ceramides and their metabolites, lactosylceramides, rise extensively in the plasma of children with chronic kidney disease, and this was associated with abnormal cardiac structure and function, suggesting an extensive role of cardiac lipotoxicity in the pathogenesis of cardiac dysfunction in the presence of kidney disease (Mitsnefes et al., 2014). Several mechanisms have been proposed for ceramides-induced cardiovascular toxicity, yet the full mechanism of action is incompletely understood. One of the suggested mechanisms is via accumulation of ceramides in myocardial cells and lead to cellular apoptosis (Parra et al., 2013). Ceramides accumulation may be attributed to an increase in nSMase without a corresponding increase in ceramidase activity (Reidy et al., 2020), or due to increased fat intake that stimulate ceramide biosynthesis (de la Maza et al., 2015). Additional evidence for the detrimental CVD outcomes associated with increased levels of circulating ceramides was provided by blocking the *de novo* pathway of ceramide synthesis in mice treated with Myriocin which showed enhanced cardiac dilation and improved cardiac contractility (Park et al., 2008).

### Vascular: Atherosclerosis and Inflammation

One of the speculated mechanisms for ceramides induced cardiovascular manifestations is that sphingomyelin, a precursor of ceramides (Figure 2), could aggregate at higher concentrations with low-density lipoproteins in atherosclerotic lesions where sphingomyelinase can also be found, suggesting a role for SMase along with ceramides in the development of atherosclerosis and coronary artery disease (Iqbal et al., 2017; Seah et al., 2020). In their observational study, De Mello et al. have found a positive rapport between plasma ceramides (C23:0 and C24:1) and inflammatory marker IL-6 (De Mello et al., 2009). Also, ceramides were reported to have stimulatory effects on TNF-α and NF-κB pathways which may work sequentially, initiating an inflammation cascade (Osorio et al., 2016; Al-Rashed et al., 2021). Eventually, those inflammatory mediators will increase the risk of developing atherosclerosis, contributing to vascular diseases (Okazaki et al., 2014; Zhang et al., 2014; Pan, 2017).

### Vascular: Oxidative Stress and Endothelial Dysfunction

**FIGURE 3:**
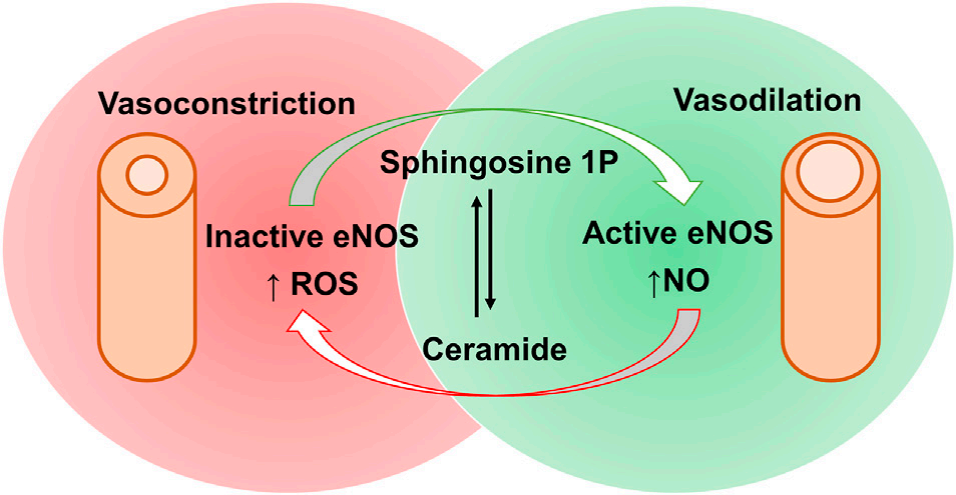
Contradictory effects of sphingosine-1 phosphate and ceramides via activation and inactivation of eNOS and the subsequent impacts on blood vessels. Sphingosine-1 phosphate causes eNOS activation, increasing nitric acid (NO) production and subsequent vasodilation. This can be opposed by ceramides mediated eNOS inactivation, increasing reactive oxygen species (ROS) production leading to vasoconstrictive effects. [eNOS, endothelial nitric oxide synthase; S1P, sphingosine-1 phosphate].

Both animal and human studies have demonstrated a positive correlation between plasma ceramide endothelium-dependent vasoconstriction being most likely the reason behind it (McGurk et al., 2021). Although it is still controversial whether ceramides produce vasodilation or vasoconstriction effects, there is a postulated mechanism for C16:0 ceramide mediated vasoconstriction through protein kinase C activation. This, in turn, increases calcium entry into vascular smooth muscles, thereby resulting in vascular contraction (Zheng et al., 2000). On the other hand, it is worthy to mention the contradictory vasodilation effects of sphingosine-1 phosphate (S1P) at low concentrations on rat aorta and mesenteric artery, which are mediated through S1P_1_ or S1P_3_ activation of endothelial nitric synthase (eNOS) and consequent release of endothelium-derived nitric oxide (NO) (Kennedy et al., 2009). Thus, the vascular tone is maintained by a balance between ceramide and S1P (Figure 3) (Van Brocklyn and Williams, 2012), which may open new routes of research for the treatment of hypertension.

**FIGURE 4:**
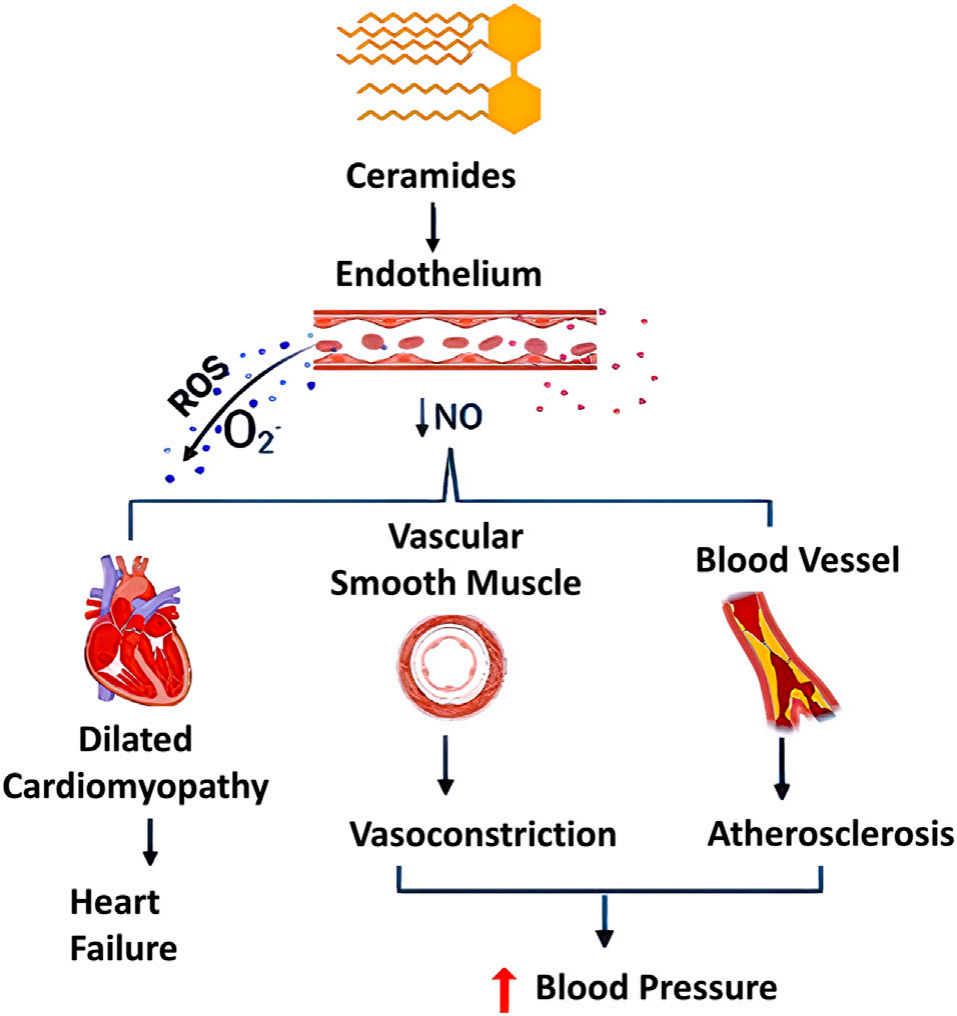
Different pathological effects of ceramides (C16:0, C18:0, and C24:1). Effects of ceramides include generation of reactive oxygen species (ROS) and decrease in nitric oxide (NO) production, adversely affecting the human body. Ceramides showed dual effect on vascular system through uncoupling endothelial nitric oxide synthase, decreasing NO availability and sustainable stimulation of ROS production especially superoxide free radicals (O_2_^.−^). This may contribute to multiple dysfunctions in the cardiovascular system including oxidative stress, atherosclerosis and it can be promoted to a dilated cardiomyopathy [NO, nitric oxide synthase; ROS, reactive oxygen species]. This figure was created using Biorender.com.

Another postulated pathological mechanism for ceramides on the cardiovascular system has involved an oxidative stress pathway through the generation of reactive oxygen species (ROS). In agreement with that context, an *in-vitro* study conducted by Akawi et al. has demonstrated that elevated levels of C16:0 ceramide not only triggers uncoupling of eNOS, but also generates ROS such as superoxide radical (O_2_^**.**−^) (Akawi et al., 2021). Consequently, a reduction of NO availability in blood vessels occurs, which can be partially explained by an increase in the activity of protein phosphatase 2A (PP2A) in vascular endothelial cells, and this may result in vasoconstrictive effects, atherogenesis, and/or oxidative stress as depicted in Figure 4.

Additionally, there has been a growing amount of evidence over the last decades that points towards a mutual synergistic relationship between ROS production and ceramide accumulation, sometimes referred to as “Feedforward Amplifying Mechanism” (Cogolludo et al., 2019). ROS involving superoxide radical, a precursor of many other free radicals, exhibit a wide range of deleterious effects on mammalian cells as they can be generated in various types of cells, including endothelial cells, aorta, and macrophages (Bhunia et al., 1997; Zhang et al., 2008). The effects of ceramides on ROS production are not limited to the activation of ROS-generating enzymes, such as NADPH oxidase and NOS; ceramides also interact with the respiratory electron transport chain and thereby increase the production of ROS as by-products (Li et al., 2010). Most notably, NADPH oxidase activity is responsible for the generation of highly reactive O_2_^**.**−^ which is unstable. Thus, it is rapidly reduced by superoxide dismutase into H_2_O_2_ that can be further reduced into another highly toxic hydroxyl radical (Das and Roychoudhury, 2014). In addition, many studies have confirmed that ceramides have the ability to induce endothelial dysfunction in small coronary arteries based on the activation of NADPH oxidase, and the consequent increase of ROS production as well as diminishing NO availability (Zhang et al., 2003; Zhang et al., 2007; Li and Zhang, 2013), As a result, this may contribute to a malfunction of coronary circulation and lead to CVDs (Figure 4). Moreover, several studies have addressed ceramides’ negative impacts on cardiac function in terms of altering signal transduction, modulation of intracellular ion channels and stimulation of apoptosis (Alewijnse et al., 2004) (Table 2).

**TABLE 2:** Summary of ceramides impacts on cardiovascular outcomes in humans.

Observed markers	Associated cardiovascular outcomes	Number of participants	References
Ceramide ratios	Major adverse cardiovascular incidents that involve acute coronary syndrome, heart failure, stroke, and CV death	N = 920 HTN patients	Yin et al. (2021)
C16:0/C24:0			
C24:1/C22:0			
C16:0, C18:0, C24:1 and Ceramide ratios: C16:0/C24:0, C18:0/C24:0, C24:1/C24:0	Cardiovascular mortality	N = 1704 CAD patients	Li et al. (2020)
C16:0, C18:0, C24:1	Cardiovascular mortality	N = 400	Targher et al. (2020)
C16:0, C18:0, C18:1	Heart failure	N = 433	Javaheri et al. (2020)
C16:0	Heart failure	N = 4,249	Lemaitre et al. (2019)
C16:0, C18:0	Hyperinsulinemia and insulin resistance	N = 2086	Lemaitre et al. (2018)
C20:0, C22:0, C24:0			
C18:0	Hyperinsulinemia and insulin resistance	N = 962	Wigger et al. (2017)
C16:0, C18:0, C24:1 and Ceramide ratios: C16:0/C24:0, C18:0/C24:0, C24:1/C24:0	Myocardial infarction, stroke, revascularization, and death	N = 495	Meeusen et al. (2018)

CAD, coronary artery disease; HTN, hypertension.

### Metabolic: Insulin Resistance and Obesity

**FIGURE 5:**
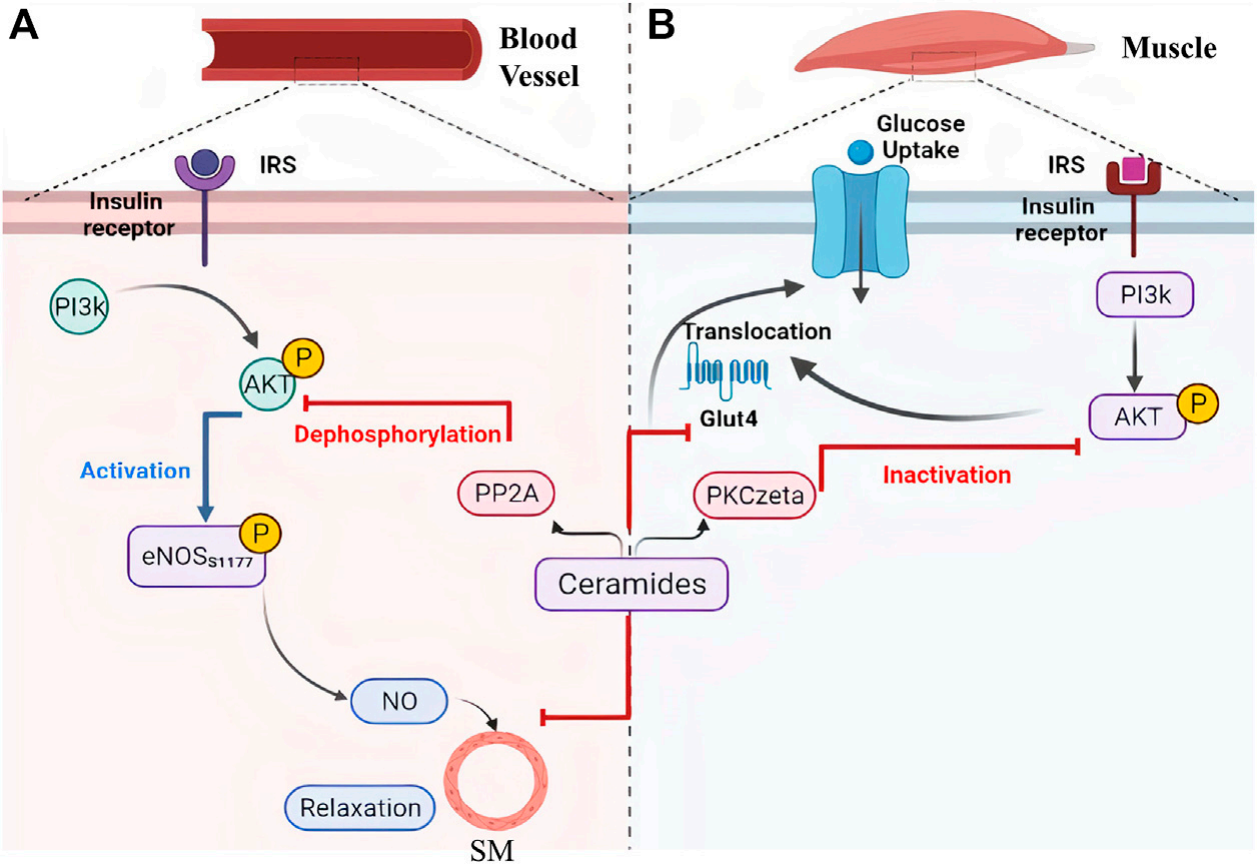
Ceramides contribute to metabolic disorders via inhibition of PI3k/Akt as a common pathway between vasoconstriction and insulin resistance. Ceramides can facilitate the inactivation of protein kinase B (AKT) through **(A)** its dephosphorylation by protein phosphatase 2A or **(B)** binding to the inhibitor PKCzeta protein which leads to narrowing of blood vessels and inhibition of glucose transporter 4 translocation (glucose intolerance), respectively. [eNOS, Endothelial Nitric Oxide Synthase; Glut 4, Glucose Transporter 4; IRS, Insulin Receptor Substrate; NO, Nitric Oxide; PI3k, Phosphoinositide-3-kinase; PKCzeta, Protein kinase C zeta; PP2A, Protein Phosphatase 2A; SM, Smooth Muscles]. This illustration was created with Biorender.com.

High saturated fat diet promote obesity, and this has been coupled with an increased risk of developing cardiomyopathy in mice models. The underlying mechanism for that was attributed to increased ceramides levels that mediated disruption of caveolae, specialized membrane invaginations important for cellular signaling, in mice heart cells (Knowles et al., 2013). It has also been shown that ceramides restrain glucose uptake by mammalian cells as a part of their role in enhancing the entry of fatty acids in adipose and non-adipose tissues (Summers, 2020). This, indeed, causes impaired glucose utilization and contributes to insulin resistance, which could be explained by two molecular mechanisms as exhibited in Figure 5. Firstly, ceramides restrict glucose transporter (GLUT-4) translocation and prevent its binding to the cell membrane. Secondly, they inactivate protein kinase B, also known as PKB/Akt, by facilitating its binding to an inhibitory protein called PKCzeta. Similarly, ceramides are also involved in the activation of PP2A, which leads to dephosphorylation of PKB/Akt (Figure 5A), and consequently suppression of its action needed for insulin signaling pathway and GLUT-4 translocation (Figure 5B) (Larsen and Tennagels, 2014). As it is known, insulin resistance is an important mechanism that is promoted by obesity and contribute not only to the development of type II diabetes but also to the increased risk of CVD related complications impacting other CVD underlying pathological mechanisms including endothelium dysfunction, constriction of blood vessels, atherosclerosis, and inflammation. To elaborate, insulin resistance alters PI3k/Akt pathway that can inhibit Akt kinase and develop an inactivated form of eNOS, in response to dephosphorylation of its serine 1177. As a result, less NO would be released from vascular endothelium, contracting vascular smooth muscles and negating insulin-mediated vasodilation effects (Huang, 2009), as shown in Figure 5A. On the contrary, defects in vascular endothelium and low NO liberation induce vasoconstrictive effects which may lead to glucose intolerance and further insulin resistance due to insufficient insulin delivery to peripheral tissues (Janus et al., 2016).

## Ceramides Targeted Therapies

**FIGURE 6:**
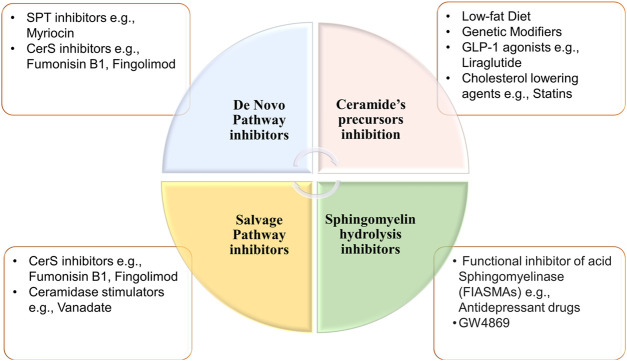
Potential inhibitors of ceramides synthesis pathways. Ceramides can be synthesized through three different pathways. First, *de novo* synthesis pathway which can be repressed by inhibiting ceramide synthases or SPT. Second sphingomyelin hydrolysis pathway that can be inhibited via functional inhibitor of acid or neutral sphingomyelinase or Ceramides can be synthesized through three different pathways. First, De novo synthesis pathway which can be repressed by inhibiting ceramide synthases or Serine palmitoyl-transferase. Second, sphingomyelin hydrolysis pathway that can be inhibited via functional inhibitor of acid or neutral sphingomyelinase. Lastly, inhibition of salvage pathway through depletion of ceramide precursors (ceramide synthases inhibition), or via increasing ceramide metabolism by ceramidases. [CerS, ceramide synthases; SPT, serine palmitoyl-transferase].

**FIGURE 7:**
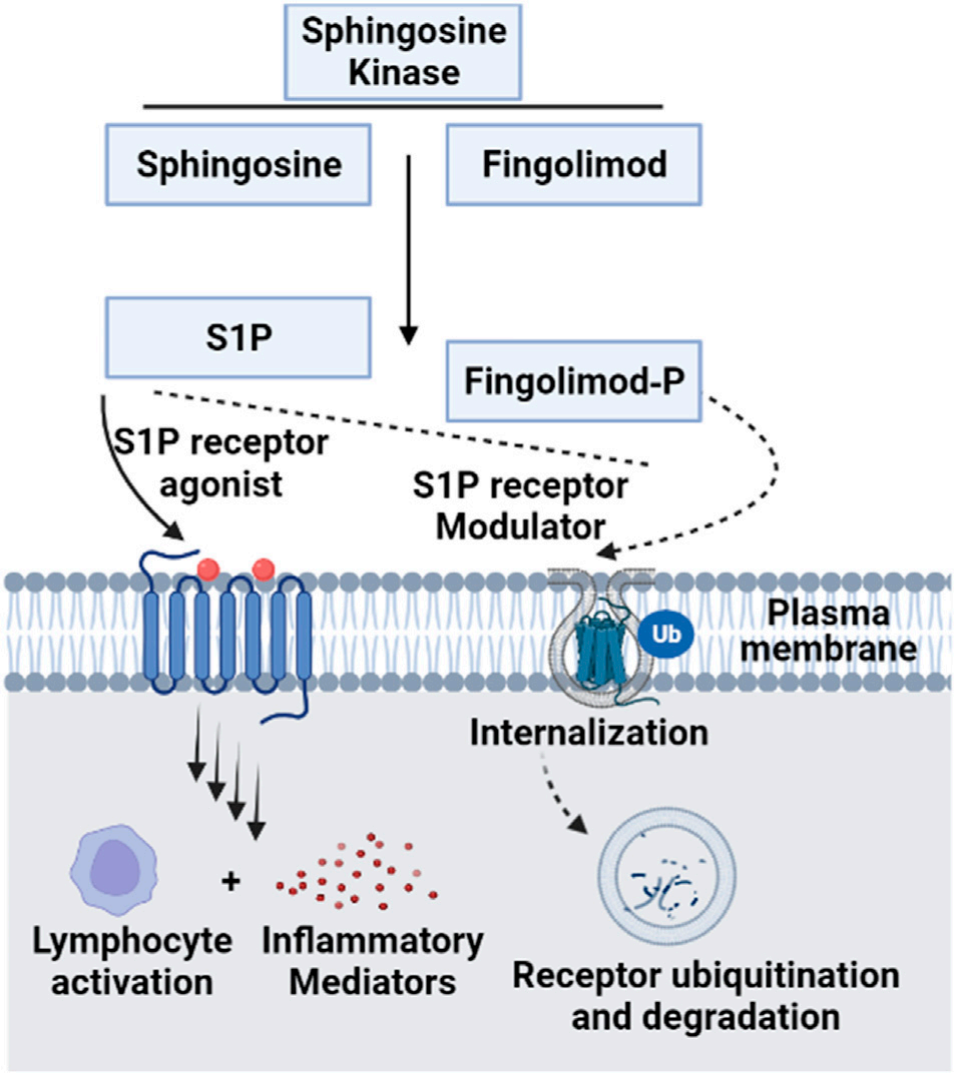
Molecular mechanism of Fingolimod as a functional antagonist for S1P receptors. Since Fingolimod structurally resembles S1P, the former can compete with Sphingosine for Sphingosine kinase and get activated by phosphorylation into Fingolimod phosphate. This active form modulates S1P receptors via internalization and ubiquitination, acting as a functional antagonist for S1P ligand on its receptors. This contributes to the regulatory functions of Fingolimod as an inhibitory of inflammatory cytokines production, which is upregulated by the disruption of S1P signaling (Van Doorn et al., 2010) [S1P, sphingosine 1 phosphate; Ub, ubiquitination]. This figure was generated using Biorender.com.

Modulation of ceramides levels and limiting their accumulation have attracted much attention recently from researchers worldwide, aiming to tackle their pathological implications and identify new therapeutic targets, especially for cardiac impairment (Mikłosz et al., 2015; Klevstig et al., 2016). Despite lacking evidence of direct cause-effect rapport between CVDs and ceramides, traditional therapies such as lipid-lowering medicines and lifestyle modifications such as low-fat diet may be employed to minimize overall risk while ceramide-specific drugs are being developed (Hilvo et al., 2020). It was shown that the HMG-CoA reductase inhibitors, or statins reduce sphingolipids concentrations such as pitavastatin treatment that lowered significantly the levels of most sphingolipids in treated cardiac patients including ceramides independently of reduction in plasma cholesterol (Meikle et al., 2015). The same effect was observed for non-statin drugs such as proprotein convertase subtilisin/kexin type 9 (PCSK9) inhibitors (Ye et al., 2020). On the other hand, activation of glucagon-like peptide 1 (GLP-1) receptor, a non-cholesterol-lowering drug, has shown cardioprotective effects against ceramides accumulation in experimental models (Monji et al., 2013). Moreover, the protective mechanism of GLP-1 receptor analogues involved suppression of the JNK signaling pathway and reduction of apoptosis induced by lipotoxicity (Leonardini et al., 2017). A prime example of GLP-1 receptor agonist is Liraglutide which is showing a promising therapeutic role in cardiovascular diseases; however, its exact mechanism is not fully understood (Marso et al., 2016). Besides its major role in controlling blood glucose level via enhancing insulin secretion (Mehta et al., 2017), a recent randomized controlled trial has proved that liraglutide can reduce body mass index, which could be partially explained by its role in reducing appetite and gastric emptying (Kelly et al., 2020). Notably, Somm et al. have inferred that liraglutide can inhibit the accumulation of C16:0 ceramide and C24:0 ceramide in methionine-choline deficient dietary mice liver and prevent subsequent inflammation and fibrosis (Somm et al., 2021). In line with these studies, liraglutide may have a potential role in reversing pathological outcomes of cardiac dysfunctions through inhibition of ceramide levels (Akawi et al., 2021). Intriguingly, various classes of drugs (listed in Table 3) were found to have multiple therapeutic effects against different diseases via targeting ceramides biosynthesis pathway as depicted in Figure 6. For example, Myriocin, SPT inhibitor, was reported to reduce atherosclerotic lesions, fatty liver progression and fibrosis induced by high ceramides levels in mice (Kasumov et al., 2015). Moreover, it could restore normal endothelium-dependent vasodilation function of blood vessels and decrease fat accumulation in diabetic rats via improving PI3K/PKB/eNOS phosphorylation and NO release which are significantly affected by any increase in ceramide levels (Chun et al., 2011). Similarly, a synthetic derivative of Myriocin called Fingolimod (FTY720) was found to inhibit ceramide biosynthesis via interfering with CerS in endothelial cells isolated from human pulmonary artery (Berdyshev et al., 2009). However, the main mechanism of FTY720 is via modulating four of the five types of S1P receptors (S1P_1_ and S1P_3–5_) (Chiba, 2020). It has become known that increased levels of S1P can evoke inflammatory outcomes through regulating lymphocyte trafficking and other inflammatory cytokines production such as, TNF-α and IL-6 (see Nagahashi et al., 2018 and references therein). Thus, S1P receptors, particularly S1P1 downregulation that occurs as a consequence of FTY720 phosphorylation may counteract the production of proinflammatory cytokines induced by the increased levels of S1P (Seki et al., 2013) (Figure 7). Due to its promising therapeutic value, researchers investigated some derivatives of Fingolimod (ST1058, ST1060 ST1072, ST1074), seeking new treatment approaches through selective ceramide reduction mechanisms. Remarkably, they found that both ST 1058 and ST 1074 could suppress CerS2, also can be inhibited by ST1060, and CerS4, whereas ST1072 selectively inhibits CerS4 and CerS6 (Schiffmann et al., 2012). Later on, Fingolimod has proved its efficacy in reversing insulin resistance via reduction of ceramide levels and enhancement of Akt phosphorylation in mice (Bruce et al., 2013). On the contrary, Fumonisin B1, a mycotoxin, retains its toxicity through inhibition of both *de novo* and salvage pathways as it can block the six isoforms of CerS (Riley and Merrill, 2019). As summarized in Table 1, several studies have investigated the role of CerS enzymes (Grösch et al., 2012) and their inhibitors as a distinct therapeutic target in the management of cardiometabolic diseases as well as other common diseases (see Choi et al., 2021 and references therein). It is noteworthy that genetic ablation of genes that encode sphingolipid biosynthesis enzymes (SPT, sphingomyelinase, ceramidases) may alleviate ceramide-associated metabolic disease (Bikman and Summers, 2011b). Ceramide levels can additionally be reduced through inhibition in the sphingomyelin hydrolysis pathway. GW4869 is commonly used as a selective repressor of nSMase, whereas functional inhibitors of aSMase such as antidepressants possess higher clinical tolerability and therefore have wider clinical applications (Kornhuber et al., 2014). Moreover, Vanadate may increase ceramide phosphorylation and metabolism via increasing ceramide kinase and ceramidase activities, respectively which will decrease ceramide concentrations in correspondence to those effects (Tada et al., 2010), Table 4 summarizes the various transgenic models for ceramide metabolism.

**TABLE 3:** Medications that target ceramides biosynthesis pathways.

Target	Drug class	Example	References
Ceramide’s precursors	GLP-1 agonists	Liraglutide	Meikle et al. (2015); Somm et al. (2021)
	Cholesterol lowering agents	Statins	
De Novo Pathway	SPT inhibitors	Myriocin	Park et al. (2008); Berdyshev et al. (2009); Riley and Merrill (2019)
	CerS inhibitors	Fumonisin B1, Fingolimod	
Sphingomyelin hydrolysis	Functional inhibitor of acid sphingomyelinase (FIASMAs)	Antidepressant drugs	Kornhuber et al. (2014)
		GW4869	
Salvage Pathway	CerS inhibitors	Fumonisin B1, Fingolimod	Berdyshev et al. (2009); Tada et al. (2010); Riley and Merrill (2019)
	Ceramidase stimulators	Vanadate	

CerS, ceramide synthase; SPT, serine palmitoyltransferase.

**TABLE 4:** Various models for ceramide metabolism.

Transgenic model	Effects	Mechanism	Ceramide’s targets	References
Mice that overexpress long-chain acyl-CoA synthetase in the heart	Initial cardiac hypertrophy and cardiac dysfunction	Lipid accumulation associated with an increase in ceramide synthesis in cardiac tissues	—	Chiu et al. (2001)
Cardiac overexpression of glycosylphosphatidylinositol -anchored human lipoprotein lipase	Dilated cardiomyopathy	Increase the *de novo* biosynthesis of ceramides and accumulation of ceramide in heart tissues	SPT inhibitors, e.g., Myriocin	Park et al. (2008)
APPL1 overexpression in transgenic mice	Showed protection against cardiac dysfunction induced by high-fat-diet	Regulation of adiponectin and insulin signaling	—	Park et al. (2013)
	Increased insulin sensitivity	Also decreased ceramide in favor of sphingomyelin biosynthesis in cardiac tissues		
Mutated (V717I) amyloid β precursor protein (AβPP) transgene in mouse hippocampus	Upregulation of ceramide synthesis in brain tissues that promote Alzheimer disease	Upregulation of ceramide synthases (increase ceramide turnover in the salvage pathway) and downregulation of sphingomyelin synthases	FTY720 counteracts reduction of sphingomyelin synthases and decrease of mRNA expression of ceramide synthases	Jęśko et al. (2020)
Transgenic mice with overexpression acid sphingomyelinase in hippocampus	Upregulation of ceramide production in the hippocampus enhanced depression-like behavior	Reduction in Akt phosphorylation at Ser473, which known to regulate neurogenesis	Functional inhibitor of acid sphingomyelinase (FIASMAs) e.g., Antidepressant drugs GW4869	Park et al. (2008); Kornhuber et al. (2014)

FTY720, fingolimod; SPT, serine palmitoyltransferase.

## Conclusion and Future Perspectives

Ceramides are endogenous lipids with various structural and biological functions that are essential to regulate myriad of cellular activities. However, high plasma levels of specific ceramides has been linked with several conditions, including CVDs, type II diabetes, obesity, hypercholesteremia, insulin resistance, and hypertension. . The pathogenesis of ceramides in cardiometabolic diseases may be partially explicated through mutual pathological mechanisms based on their inflammatory, and oxidative stress effects in addition to being the main players in the dysregulation of the PI3k/Akt pathway. Hence, targeted inhibition of ceramides biosynthesis may broaden the scope of non-invasive therapies for these diseases. This, indeed, needs further studies to fully understand the role of ceramides and their pathological mechanism of actions. Additionally, more research is needed to screen the derivatives of available drugs that can modulate ceramide pathways, hoping to discover more selective and efficient treatments.
